# When narrative practice suddenly goes online due to COVID-19 …

**DOI:** 10.1177/1473325020981086

**Published:** 2021-03

**Authors:** Chitat Chan, Hoyee Au-Yueng

**Affiliations:** Department of Applied Social Sciences, The Hong Kong Polytechnic University, Kowloon, Hong Kong

**Keywords:** Identity, media, technology, narrative practice, digital storytelling

## Abstract

This article is a reflective consolidation of our practice experience in Hong Kong during the COVID-19 period, in which social work educators and practitioners needed to work online in a prolonged period of social distancing. It illustrates in what ways online practices may denote emerging knowledge and skills that are worth further discussion. These reflections have been consolidated as four knowledge/skill domains in our afterthoughts: i) Context, ii) Conversation, iii) Communication-Modality, and iv) Circulation. These insights may inspire social work educators and practitioners to comprehend the potential of media technologies more fully.

## The evolution of a training activity

Narrative practice (NP) is an approach used in social work that helps participants narrate life stories and reconstruct dominant storylines, which can enable individuals to explore their unique wisdom so they can address life’s challenges more successfully (Chan et al., 2020; White, 2007). We are staff from a social work department at The Hong Kong Polytechnic University (PolyU). Since the end of 2019, we have run an after-lunch social gathering, *Genuine Café,* that demonstrates narrative practice and let trainees practice NP (https://www.humans.asia/web/organisation-details.php?lang=en&organisation_id=258). In each Genuine Café session, the host uses NP skills to conduct an interview, and invite other participants to provide feedback and share their own stories. Skills used in the session are explained and discussed in the latter part of the program, enabling participants to learn from the interview process. While this is a usual internal training program, this unusual coronavirus period made our usual practice public and became unusual.

In 2020, the single issue that drew attention from all around the world was definitely the coronavirus disease 2019 (COVID-19) (BBC News, 2020; The Visual and Data Journalism Team, 2020). Due to the lockdown and quarantine policies, we were forced to fast-forward the ability to teach online. Initially, we planned to meet on campus in October 2019, but we could not make it because of the severe destruction of the PolyU campus caused by violent conflicts between social protestors and the police in the large-scale anti-extradition bill protests in Hong Kong (BBC News, 2019; Grundy, 2019). We hosted our first few Genuine Café sessions in a restaurant with a small group of eight to ten people. After a few sessions, we came back to host the event on campus, and our guests could choose to be interviewed on campus or online. However, this format did not last long as the pandemic spread to Hong Kong, which called for a stop in all face-to-face events on campus. Thus, the Genuine Café moved from a semi-online format to a completely online format since March 2020. It is now a weekly online event, and there were occasions that we had over 80 participants in a single session.

News media typically reports confirmed cases and death, and people usually tend to focus on difficulties, obstacles, and problems brought by COVID-19. Nonetheless, are there opportunities and possibilities offered by this global pandemic? Throughout the evolution of Genuine Café from its initial after-lunch chitchat mode to its current online mode, we have encountered different hurdles and experimented with new methods. These experiences have inspired us to ponder what has been made possible with the use of communication technologies. This article is a reflective consolidation of our experience in that period of time.

### Episode 1: Face-to-“screen” communication, do we know how to handle?

**Figure 1.**
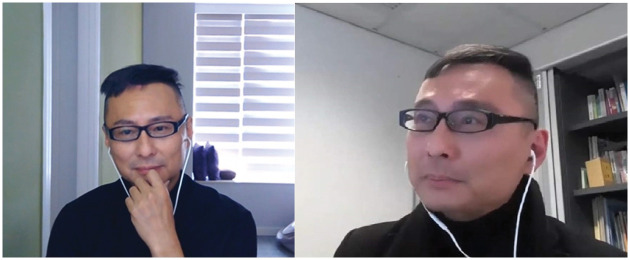
Screenshots from different camera angels.

Social work practice traditionally relied on face-to-face communication. In Genuine Café, we were suddenly forced to communicate via an online platform due to the pandemic. Aside from affecting your appearance, camera angles and framing methods may make it more apparent as to whether or not you are paying enough attention to communicate with your service users (see Figure 1). We learned that camera angles make a difference in how we maintain eye-contact with interviewees during online communications. We noticed that face-to-screen communication is a very different form of communication, and traditional social work training has not taught us these skills and knowledge.

### Episode 2: Blurred boundaries of new opportunities?

Cho was one of the Genuine Café guests after the program went online completely (https://www.humans.asia/web/video-play.php?video_id=49). She shared her story about her boyfriend, including the discovery of his affair with his ex-girlfriend, and how she subsequently struggled with her relationship with him. Stories related to love affairs are often sensitive, especially within a small professional circle in a small place like Hong Kong. Usually, it is not easy to have practitioners from the social service sector willing to share their love problems online, and that online interviews always trigger us to query what sort of consent and boundary we should set. Online communications can mean a blurred private/public boundary, but it also creates an environment that a participant can keep his/her identity remains anonymous in a real time conversation. Before the event, Cho was fully informed about the event arrangements, such as the event would be video recorded with participants present, and informed consent was obtained. Cho ultimately chose to share her story online.

### Episode 3: Connection beyond geographical boundaries?

South Korea was one of the first countries with an outbreak of COVID-19. Fanny is from Hong Kong, and she was studying and working in South Korea during the outbreak (https://www.humans.asia/web/video-play.php?video_id=34). Fanny was the first in many overseas guests we interviewed. With technology, we overcame geographical boundaries. In addition, technology allowed Fanny to share photos showing real time situations from South Korea, which helped event participants understand her story more comprehensively despite conversing remotely.

### Episode 4: What can we do with colors and shapes in our worker-client communications?

**Figure 2.**
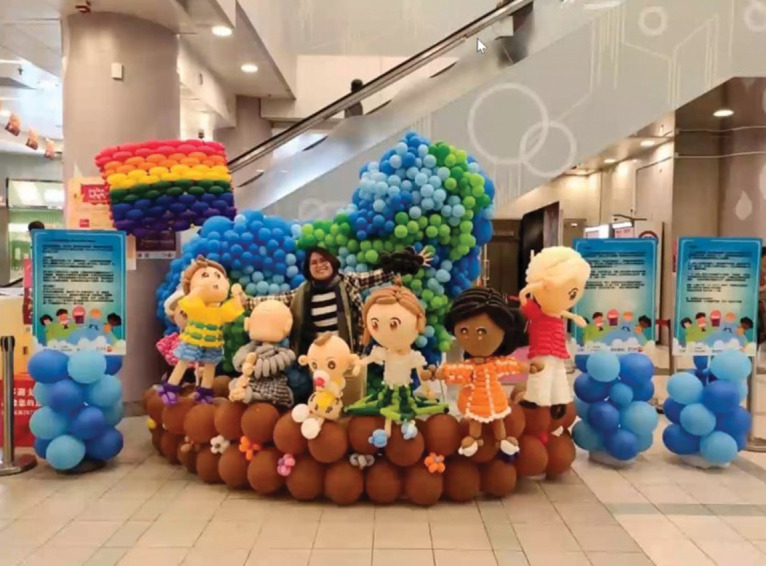
▪▪▪.

Chocolate shared in Genuine Café about her journey in becoming a full-time balloon artist (https://www.humans.asia/web/video-play.php?video_id=48). Similar to other forms of art, it is not easy for others to imagine how the art looks like, and especially in Chocolate’s case, considering balloon art is not common in Hong Kong. In verbal communications, visuals might not be fully described easily. During Chocolate’s interview, she shared with participants samples of her work in photographic form (see Figure 2). They turned out to be extraordinarily big—as big as a room, not something that you can hold with hands—thus also difficult for her to display with us in person. If we could not see these artworks in pictures, we could hardly understand the impact and social meaning of her works. Visual presentations allow us to ask questions about colors and forms. Moreover, her answers reflect her values, visions, and plans for the future. Visual representations expand our understanding and enable interviewers to ask questions based on those visuals.

### Episode 5: Is this also an opportunity for service users to reach a broader audience?

Eli is a student from a university in Hong Kong (https://www.humans.asia/web/video-play.php?video_id=36). In her interview, she talked about her eye disease and because of her bad eyesight, it was difficult for her to look at the projectors when she attended class in person. In attending class in a physical classroom, she needs to use a magnifying lens to help her see the lecture notes. Due to the pandemic, all teaching and learning went online. Online learning enables Eli to look at her own computer screen, which she can adjust the scale to facilitate better reading. In Eli’s sharing, she shared her wisdom on how she overcame her illness and learning difficulties. Since we found her story inspiring and meaningful, we made a story page on our project website (https://www.humans.asia/web/book-intro.php?lang=hk&book_id=220). This story page shows the segments of Eli’s story and photos that she wants to show to others. The website has a “make an appointment” button that enables groups and individuals to meet with Eli and share her story in their contexts. That is, besides the interview, the website enables Eli and her story to reach a broader audience and connect people.

### Episode 6: Enabling a mutual support community?

Doris had health problems that affected her performance in a public examination (https://www.humans.asia/web/video-play.php?video_id = 50). Doris believes that she studied too hard for the exam, and her health was severely impacted. After she recovered from her health problems, she had many occasions in different contexts to share her story to remind other students to take care of their health and avoid studying too hard. Doris never understood her impacts on others until she came across a Genuine Café participant who attended one of her talks previously. In Genuine Café, the host asks participants about which part of the story inspires them, and if the participants have any relevant experiences. A participant took this opportunity to thank Doris for her sharing some years ago, and that her sharing has impacted the participant’s view about studying and health. Through the feedback from this participant, Doris felt encouraged and reconnected with the people she met before. This reconnection surprised us. Online communications enable us to build a mutually supportive community, in which protagonists can connect or reconnect with people they want to meet (or have already met).

### Episode 7: Does it mean a stronger visualization and reviewability?

**Figure 3.**
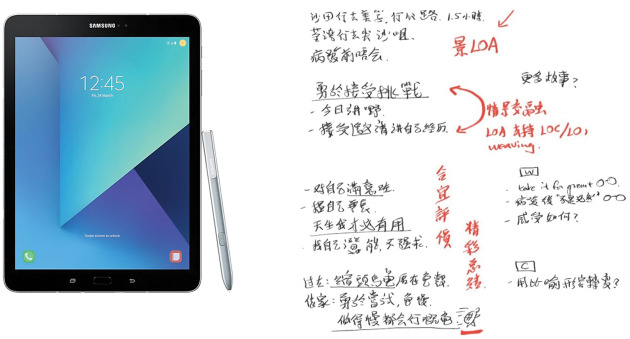
▪▪▪.

Apart from sharing and learning from stories, Genuine Café also aims to provide opportunities for practitioners to practice NP-based interviewing skills. At the end of each Genuine Café session, the host and our colleagues would analyze the interviewing process. One of our colleagues uses an electronic notepad to help him remember and organize the conversation effectively (see Figure 3). In offline settings, we did not have a chance to see how he organizes those conversations. However, online communications enabled us to see his notes and understand the process collectively as a team. He was able to show all of his notes and drawings immediately after the session from a remote site. Online communications have limitations, but they also enable us to have stronger visualization and multimedia communication. Moreover, we also record and review interview processes online, and share our analysis spontaneously with the participants.

## Afterthoughts

These episodes denote emerging knowledge and skills that have been triggered by the new environment of communication. Here, we summarize our reflections related to narrative practice in the new environment of communication using four Cs – Context, Conversation, Communication-modality, and Circulation.

First, is it possible for social work practitioners to utilize avenues of communication to facilitate their initial engagements with service users? The *Context* domain refers to strategic considerations of technological, sociocultural, institutional, and psychosocial conditions that precede and follow a communication, which influence the meaning and effect of that communication. There are practices and research closely related to this domain (Chan, 2006; Dahya, 2017; Kedzior and Allen, 2016). Skill sets covered by this domain include impression management, initial engagement, and setting expectations and boundaries. Genuine Café positions the protagonist as someone sharing insights rather than a client seeking help, while other participants position themselves more as active dialogue partners than passive audiences.

Second, is it possible to utilize the new media environment to facilitate spoken conversations? The *Conversation* domain refers to the use of spoken or written dialogues in a contemporary media environment to provide guidance with focused inquiries at an individual, group, or community level. There are practice research studies that can inform the development of this domain (Chan and Holosko, 2017; Chan and Ngai, 2019; Leung et al., 2017). Skill sets covered by this domain include counseling skills, interview skills, reflective questioning skills, and how to set up synchronous distance communication. In Genuine Café, the host interviews a voluntary guest, and invites other online participants to respond to the guest’s story. The host applies narrative practice skills, and commentators can also fully utilize online tools to explicate the conversation process and provide feedback (see Episodes 2, 3).

Third, is it possible to go beyond spoken or written conversations and address practice opportunities offered by multimedia? The *Communication-Modality* domain is closely related to the Conversation domain, but it goes beyond spoken and written dialogues and addresses the limitations and possibilities offered by multimodality, which considers communication practices in terms of textual, aural, linguistic, spatial, and visual resources (Chan et al., 2012; Chan and Sage, 2019; Chan and Yau, 2019; Kress, 2009). Skill sets covered by this domain include decoding and encoding messages in different modalities and using multimedia, genres, symbols, and slang. In Genuine Café, participants, including the guest, can opt to be anonymous or known, and the platform allows guests and hosts to share images, music, as well as handwriting (see Episodes 1, 4, 7).

Fourth, is it possible to utilize the new media environment to produce and circulate preferred narratives and build communities? The *Circulation* domain covers knowledge and skills beyond synchronous communications. It is related to producing and circulating a preferred narrative in a contemporary media environment as well as discussions about media literacy, which refers to the ability to access, understand, and create communications in a variety of contexts (Buckingham, 2003; Chan, 2019; Chan and Holosko, 2018; Lee, 1999; Ofcom, 2004). Skill sets covered by this domain include identity presentations, online advocacy, analyzing audiences’ online behaviors and characteristics, building online communities, knowledge about laws and regulations related to data privacy, and online media distribution. In Genuine Café, recorded interview sessions can be uploaded online, and practitioners and protagonists can work together to further develop story pages with detailed contents and references (see Episodes 5, 6).

Social work researchers have long been advocating the use of information and communication technology (ICT) to facilitate training and practice (Chan and Holosko, 2016; Hill and Shaw, 2011; Ramsey and Montgomery, 2014; Wretman and Macy, 2016), but it is in this critical period that we were really pushed to fully utilize online communication technology. We have witnessed how it has changed the ways we implement narrative-based interventions and enabled service users to distribute their preferred narratives. These knowledge/skill domains illustrated in this article are far from comprehensive, but they open a range of possibilities. The potential of new technology can only be further developed if practitioners take an informed position in using those technologies. There is a need for interdisciplinary collaboration—requiring partnerships with technologists, human service practitioners, and media studies researchers. These cross-sector partnerships may create changes that extend beyond a field and open an underdeveloped research area.
